# Experimental human hookworm infection: a narrative historical review

**DOI:** 10.1371/journal.pntd.0009908

**Published:** 2021-12-09

**Authors:** Paul R. Chapman, Paul Giacomin, Alex Loukas, James S. McCarthy

**Affiliations:** 1 Clinical Tropical Medicine, QIMR Berghofer Medical Research Institute, Herston, Australia; 2 Infectious Diseases Unit, Royal Brisbane and Women’s Hospital, Herston, Australia; 3 Centre for Molecular Therapeutics, Australian Institute of Tropical Health and Medicine, James Cook University, Cairns, Australia; Wellcome Trust Sanger Institute, UNITED KINGDOM

## Abstract

In 1896, a serendipitous laboratory accident led to the understanding that hookworms propagate infection by penetrating skin, a theory that was then confirmed with the first experimental human infection, reported in 1901. Experimental human infections undertaken in the 20th century enabled understanding of the natural history of infection and the immune response. More recently, experimental hookworm infection has been performed to investigate the immunomodulatory potential of hookworm infection and for the evaluation of hookworm vaccines and chemotherapeutic interventions. Experimental human hookworm infection has been proven to be safe, with no deaths observed in over 500 participants (although early reports predate systematic adverse event reporting) and no serious adverse events described in over 200 participants enrolled in contemporary clinical trials. While experimental human hookworm infection holds significant promise, as both a challenge model for testing anti-hookworm therapies and for treating various diseases of modernity, there are many challenges that present. These challenges include preparation and storage of larvae, which has not significantly changed since Harada and Mori first described their coproculture method in 1955. In vitro methods of hookworm larval culture, storage, and the development of meaningful potency or release assays are required. Surrogate markers of intestinal infection intensity are required because faecal egg counts or hookworm faecal DNA intensity lack the fidelity required for exploration of hookworm infection as a vaccine/drug testing platform or as a regulated therapy.

## Introduction

Hookworms are soil-transmitted helminths whose adult stage reside in the small intestine of their hosts where they feed on blood. Globally, an estimated 450 million people have chronic hookworm infection, which results in an estimated 2.1 million disability-adjusted life years lost and accounts for over US$100 billion in global economic losses [1].

Human infection is caused primarily by 2 human-specific species of hookworm, *Necator americanus* and *Ancylostoma duodenale*, and a third species, *Ancylostoma ceylanicum*, is adapted to humans as well as to cats and dogs and is found in Southeast Asia and the Pacific. Ground itch and Wakana syndrome are well-described syndromes associated with hookworm infection. Ground itch is the pruritic dermal reaction produced on dermal penetration by hookworm larvae, while the Wakana syndrome describes a dry irritable cough associated with penetration of upper airways. Arrival of adult worms in the intestine is, in some cases, associated with gastrointestinal symptoms such as epigastric discomfort, nausea, flatulence, and early satiety. To feed on erythrocytes, adult hookworms damage the small intestinal mucosa, causing mucosal haemorrhage. High intensity infection, especially with *A*. *duodenale*, may lead to iron deficiency anaemia (IDA) as a result of excess blood loss in susceptible individual. This is the principal disease manifestation of chronic hookworm infection [2].

Some aspects of hookworm biology, such as larval maturation in various media [3,4], larval motility, the production of various excretory/secretory (ES) products [5], and tissue penetration [6] can be studied in vitro. However, an in vitro model that permits development of adult worms from eggs or propagation of adults does not exist.

Hookworms are highly host specific, limiting the use of animal models to either their natural host with their naturally infecting parasites (e.g., dogs and *Ancylostoma caninum*), or to a small number of permissive laboratory hosts (e.g., hamsters and *N*. *americanus*) in which partial immunity develops, limiting extrapolation of results to human infection [7]. Although observing natural human infection allows appropriate evaluation of the immune responses to parasites, such studies are confounded by factors such as the exposure history of individual study subjects, nutritional status, and polyparasitism.

Human challenge studies allow prospective observation of host–pathogen interactions, the development of immunity, and estimation of therapeutic effect in a controlled environment. Ethical and regulatory frameworks for human challenge experiments have been developed [8], and contemporary experimental human infection studies have been performed with a wide range of human pathogens, including *Helicobacter pylori* [9], *Plasmodium* spp. [10], *Salmonella* Typhi [11], *Vibrio cholerae* [12], *Schistosoma mansoni* [13], influenza virus [14], respiratory syncytial virus [15], human coronaviruses [16], and human hookworms. The purpose of this review is to collate historical aspects of experimental hookworm infection, the methodologies for conducting these experiments, clinical end points, and safety outcomes.

## Methodology

A 2-step search strategy was employed to retrieve publications in any language that detailed experimental infection of humans with human hookworm larvae (*A*. *duodenale*, *A*. *ceylanicum*, or *N*. *americanus*). Firstly, the PubMed database was interrogated using the following Boolean operations: ((Hookworm[tiab] OR Hookworms[tiab] OR Ancylostoma duodenale OR Ancylostoma ceylanicum OR Necator americanus OR “ancylostomatoidea”[MeSH Terms] OR “Hookworm Infections”[Mesh]) AND (Experimental infection OR (experimental[tiab] AND infection[tiab]) OR volunteer* OR Inoculation OR Therapeutic infection[tiab] OR iatrogenic hookworm infection[tiab])). Bibliographies of the included studies were then reviewed and further publications sourced.

Manuscripts that contributed information regarding laboratory manufacture of hookworm larvae and their use in experimental infection, that detailed adverse events or reactions, or described the measurement of relevant clinical end points were reviewed in detail. As reporting of these data was limited and inconsistent, a systematic appraisal of these issues was not possible. This review therefore discusses the experience of experimental human hookworm infection to date and outlines the challenges and opportunities ahead in using this system.

## Results

**Fig 1:**
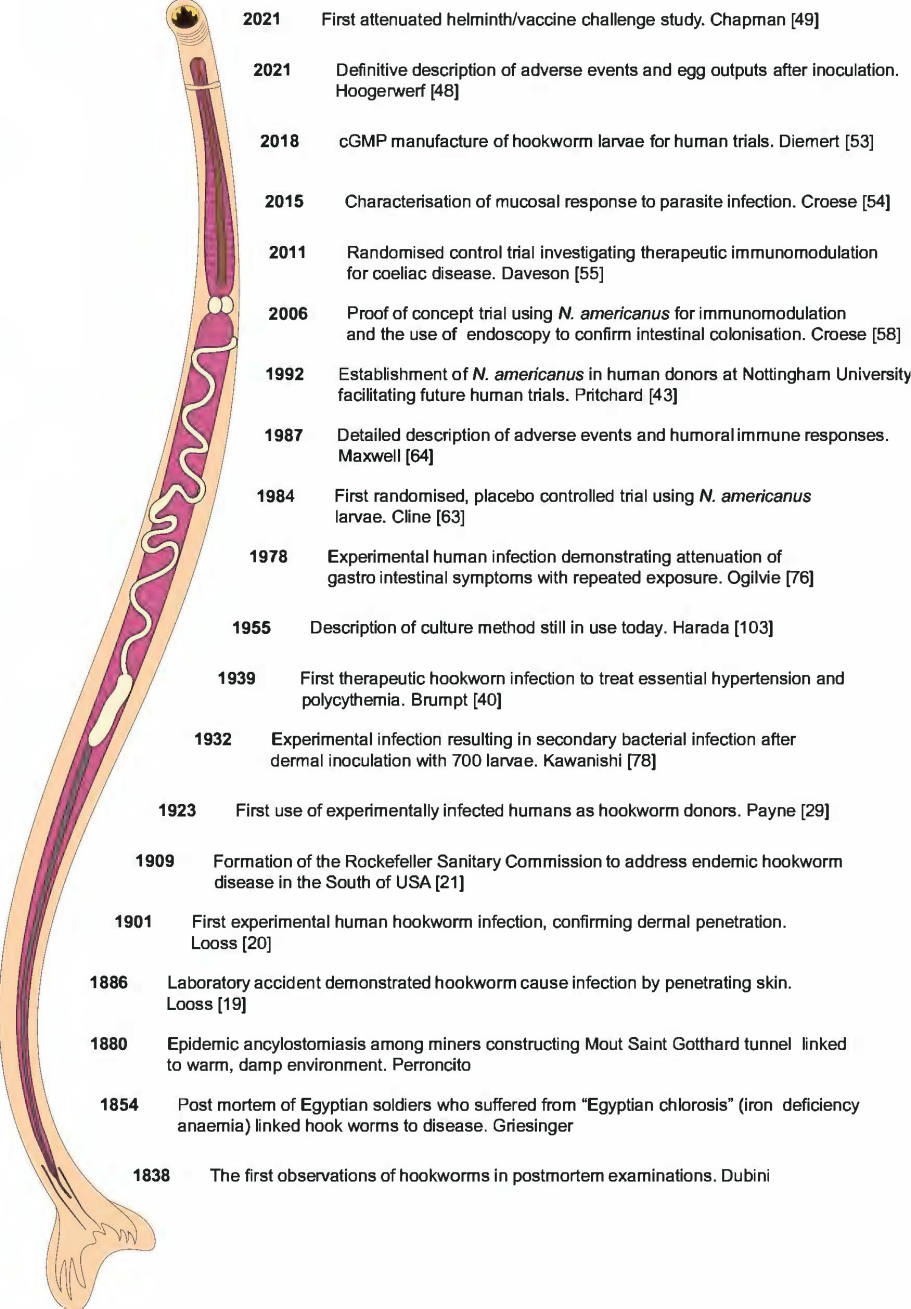
Significant events in experimental human hookworm infection. Historical aspects of human hookworm infection.

Significant events in experimental human hookworm infection are summarised in Fig 1.

While the Papyrus Ebers (1550 BC) probably refers to round worms (*Ascaris lumbricoides)*, tape worms (*Taenia* spp.), and Guinea worm (*Dracunculus medinensis*), there is controversy as to whether ancient Egyptians knew of hookworm disease [17]. Francis Cox, in 2004 [18], eloquently reviewed the contemporary history of human hookworm and other parasitic diseases; the salient points are briefly summarised here. The first documented observations of human hookworm were in postmortem examinations by Dr. Angelo Dubini in 1838. The connection between human disease and hookworm infection was demonstrated by Wilhelm Griesinger in 1854 based on postmortem observations of hookworm infection in a soldier who had apparently died of diarrhoea and suffered from “Egyptian chlorosis” (IDA). Through detailed microscopic studies in 1880, Edoardo Perroncito established that ancylostomiasis was endemic among miners constructing the Mount Saint Gotthard tunnel in Switzerland. In 1896, a serendipitous laboratory accident, in which Arthur Looss spilt hookworm larvae on his hand, led to the discovery that hookworm infection is, in fact, propagated through penetration of intact skin [19]. He then confirmed this theory by dermally applying hookworm larvae to the leg of a hospitalised patient awaiting amputation of the limb. Microscopic and histopathologic examination of the amputated limb revealed penetration of the skin by the larvae [20].

Hookworm disease was considered to pose such a public health risk in the United States of America that, in 1909, the Rockefeller Sanitary Commission (RSC) was formed to address the widespread hookworm disease in the southern states, with the stated goal “to bring about a co-operative movement of the medical profession, public health officials, boards of trade, churches, schools, the press and other agencies for the cure and prevention of hookworm disease” [21].

The success of the RSC provided the model for future public health works of the Rockefeller Foundation (RF), and its associated organisations including the International Health Division (IHD), the legacy of which influenced the establishment of the World Health Organization (WHO) [22].

### Experimental human hookworm infection in the 19th and 20th centuries

In the included 40 publications, more than 599 individuals have participated in experimental human hookworm infection (S1 Table). As noted previously, the first experimental human infection conducted by Looss and reported in 1901 proved that hookworm infection occurs via dermal penetration of larvae [20]. This finding was quickly confirmed by Bentley, in his role as the medical officer to the Empire of India and Ceylon Tea Company, when he experimentally reproduced ground itch in Indian tea labourers by dermally applying soil contaminated with human faeces [23].

Experimental human infection studies undertaken in the early 20th century in the USA, Puerto Rico, India, China, South Korea, Thailand, and Japan increased understanding of the natural history of human hookworm infection. Observations included the ability of *A*. *duodenale* to produce patent infection after oral inoculation, while *N*. *americanus* required dermal contact of larvae [24–31].

In 1923, Payne demonstrated that larval infectivity declines with larval age and that *N*. *americanus* is more efficient than *A*. *duodenale* at producing patent infection via dermal penetration [29], a finding confirmed by Svensson in 1927 [30] and again by Mizuno and Yanagisawa [28], the latter in meticulously conducted experiments where dermal application of *N*. *americanus* or *A*. *duodenale* larvae were compared. These experiments demonstrated that *N*. *americanus* larvae were more than 3 times as likely to survive to maturity. Additionally, Payne reported concomitant immunity whereby “a prior infestation may have some influence in rendering the establishment of a new infestation more difficult,” a finding that was eventually corroborated by endoscopy in 2006 [32].

The remarkable efficiency of *N*. *americanus* larvae to successfully invade was demonstrated by Beaver in 1955 where 9 participants (8 hookworm naive and 1 previously treated) were exposed via dermal application to exactly 3 larvae [33]. All subjects experienced a pruritic lesion at the inoculation site, with individually discernible penetration sites in most cases. Interestingly, the skin reaction was most severe in the subject who had previously been exposed to hookworms. A 5 of the 9 subjects developed patent infection, illustrating the extraordinary host adaptation and efficiency of *N*. *americanus* infection.

Cutaneous larval migrans (CLM) is caused by dermal infection with zoonotic helminth larvae, including the dog and cat hookworm *Ancylostoma braziliense*. In the early 20th century, it was noted that CLM due to exposure *A*. *braziliense* did not occur in Africa or the Indian subcontinent, despite the apparent presence of the relevant hookworm species. Two independent, yet parallel experiments, involving dermal application of *A*. *braziliense* larvae in Texas [34] and India [35] confirmed this clinical observation. The medical student participants in Texas suffered severe CLM; conversely, the participants in India suffered no discernible dermal reaction. These differences prompted a detailed description of subtle anatomical differences of isolates by Biocca, leading to the acceptance that there were separate species, namely *A*. *braziliense* (cat and dog hookworm) and *A*. *ceylanicum (*cat, dog, and human adapted) [36]. Repeated experimental infections with *A*. *ceylanicum* have failed to produce CLM, demonstrating the host adaption of this species [24,37,38] and establishing *A*. *ceylanicum* as the third hookworm species that could complete its life cycle in humans.

The first therapeutic human infections with hookworm were performed in 1939 for the treatment of polycythemia. Successful normalisation of the erythrocyte count was achieved through the induction of iron deficiency by dermal application of 300 *A*. *duodenale* larvae [39]. The authors subsequently reported their experiences in undertaking therapeutic infection of 53 patients suffering from polycythaemia or hypertension [40]. These infections were performed with an average of 400 *A*. *duodenale* larvae.

### Experimental human hookworm infection in the modern era

There have been 244 individuals inoculated with *N*. *americanus* larvae since the first randomised clinical trial performed by Cline and colleagues in 1984 (Table 1). All but 2 of these trials (Wright and Bickle [41] and Geiger and colleagues [42]) have been facilitated by the use of larvae that originate from a line of *N*. *americanus* originally sourced from Kar Kar Island, Papua New Guinea and initially maintained in human donors at Nottingham University [43]. This has facilitated several trials in the United Kingdom [44–48], as well as propagated hookworm infections in human donors in Australia, the USA, and the Netherlands, which have since been the source of larvae used in published trials [49–54]. Experimentation with *A*. *duodenale* in the modern era has been conspicuously absent, perhaps in part due to the pioneering work at Nottingham university to establish a Necator model and perhaps due to safety concerns from the greater blood loss in *A*. *duodenale* infection compared to *N*. *americanus* [2].

**Table 1:** Inoculation characteristics and adverse events reported in contemporary clinical trials of experimental infection with *N*. *americanus* larvae.

Reference	Infection characteristics			Dermal symptoms			Gastroenterological symptoms				Patency infection (% exposed)
	Inoculum Dose/number L3 (cumulative dose)	Population	No of subjects	Description	Severity	Duration (days)	Description (number)	Severity (number)	Peak (duration)	Rescue medication (number participants required)	(Microscopy or molecular)
**Cline (1984) [63]**	1/45 (45)	HNV	29	Maculopapular rash	NR	NR	Diarrhoea, flatulence, nausea	Mild to moderate	29–38 (28–EOS)	0	100% Mi
**Maxwell (1987) [64]**	1/50 (50)	HNV	5	Papular rash, intensely pruritic	NR	1–6	Flatulence, pain, nausea	Mild moderate(3), mod severe(1), severe(1)	NR (30–45)	1	100% Mi
**Wright (2005) [41]**	2/50 (100)	HNV	1	Itchy papular rash	NR	47	Mild nausea, moderate pain	Mild moderate with first infection, not present in second	NR (26–45)	0	100% Mi
**Croese (2006) [58]**	1–2/25–100/50–100*	HNV + HN+Cd	12	Mild itch/pruritic rash	Mild	14	NR	NR	NR(NR)	0	100% Mi
**Mortimer (2006) [47]**	1/10–100/10–100*	HNV	10	Maculopapular rash/severe rash (100L3)	Mild (9)/severe (1)		Pain, diarrhoea	Mild 9/severe1 (100L3)	21–60 (21–EOS)	1	100% Mi
**Geiger (2008) [42]**	1/50 (50)	HNV	2	Pruritic rash	Mild	22	Pain/flatulence	Mild	NR (46–64)	0	100% Mi
**Feary (2009) [46]**	1/10 (10)*	HN+AR	14	Skin itching and redness	Mild	1–7	Indigestion (13), pain (1)	Mild (13)/severe (1)	NR(NR)	1	69% Mi
**Feary (2010) [45]**	1/10 (10)*	HN+A	17	Skin redness and itch	Mild	21	Abdominal pain	Mild 16/severe 1	29–112 (29–EOS)	1	56% Mi
**Daveson (2011) [65]**	2/5–10 (15)*	HN+CeD	10	Pruritus, papules	NR	28	Pain/flatulence	NR	NR (21–112)	0	50% Mi
**Croese (2015) [54]**	2/10 (20)*	HI + CeD	12	Blistering (1/12)	NR	3	Intermittent colic (1/12)	NR	NR	0	100% Mo
**Diemert (2018) [53]**	1/25–50 (25–50)*	HNV	20	Pruritic rash	Mild to moderate	Med 26 (4–69)	Flatulence, bloating, pain	Mild to moderate	28–35 (21–84)	0	40%–90% Mi + Mo (25 versus 50 L3)
**Hoogerwerf (2019) [52]**	1/50 (50)^#^*	HNV	4	Pruritic rash	Mild	11–32	Pain, nausea, flatulence	Mild to severe	NR (21–63)	0	100% Mi+Mo
**Croese (2020) [51]**	2/10–20, (20–40)*	HN+CeD	47	NR	NR	NR	Pain, flatulence, nausea	Mild	NR (NR)	2	77% Mo
**Tanasecu (2020) [44]**	1/25 (25)*	HN+MS	35	Skin reaction	NR	NR	NS	NR	NR	0	66% Mo
**Hoogerwerf (2021) [48]**	1–3/50 (50,100,150)^#^*	HNV	23	Pruritic rash	NR	Med 34 (0–77)	Pain, nausea, flatulence	Mild to severe	28–35 (0–125)	3	100% Mi + Mo
**Chapman (2021) [49]**	1/30 (30)^#^*	HNV	15	Pruritic rash, 226mm2 IQR 110–263	Mild	Med 2 IQR 1–3	NS	Mild	NR	0	100% Mo
**Total participants inoculated with *N*. *americanus* larvae**			244				Total participants requiring rescue medication for GI AE			9	

^#^ Inoculum divided and applied across several areas simultaneously.

* Inoculum derived from Kar Kar Island isolate, originally maintained in human donors at Nottingham University.

EOS, end of study; HI+CeD, hookworm-infected coeliac disease; HN+A, hookworm-naive asthma; HN+AR, hookworm-naive allergic rhinoconjunctivitis; HN+Cd, hookworm-naive Crohn disease; HN+CeD, hookworm-naive coeliac disease; HN+MS, hookworm-naive with multiple sclerosis; HNV, hookworm-naive volunteer; IQR, interquartile range; Med, median; Mi, microscopy; Mo, molecular; NR, not reported; NS, not significant compared to placebo.

The observation that allergy and inflammatory diseases are more common in developed countries than developing countries has led to investigation of the immunomodulatory potential of hookworm infection [55]. *N*. *americanus* infected individuals produce higher levels of the regulatory cytokine IL-10 and impaired production of pro-inflammatory IFN*γ*, IL-5 and IL-3 [56]. Use of hookworm infection as a novel therapeutic in inflammatory bowel disease [57], coeliac disease [50,53,54,58], allergic airway reactivity [46], asthma [45], and multiple sclerosis [44] has been published, with further clinical trials registered for metabolic disease [59], ulcerative colitis “Hookworm therapy for maintenance in ulcerative colitis: A placebo-controlled pilot study investigating the feasibility and efficacy of hookworm inoculation in patients with ulcerative colitis currently in remission” [60], and cancer therapeutics “Hookworm Therapy for young people at high risk for colorectal cancer”[61].

Human challenge studies provide a gold standard approach for testing of therapeutics including subunit hookworm vaccines. It is important to note that results of challenge studies undertaken in healthy hookworm-naive human volunteers may not be generalisable to populations where hookworm disease is endemic and polyparasitism is common. Building on historical data in animal models of attenuated hookworm vaccine studies [64], the first helminth challenge study to assess the efficacy of an attenuated *N*. *americanus* larvae vaccine in humans has recently been reported [49], and several challenge studies have been registered “Efficacy of Na-GST-1/Alhydrogel Hookworm Vaccine Assessed by Controlled Challenge Infection” [65] and “Immunisation, Treatment and Controlled Human Hookworm Infection (ITCHHI)” [66].

A yet to be explored approach is the use of transgenic helminths, such as hookworms, to secrete therapeutic moieties. With the advent of genome editing tools, such as CRISPR/Cas-9 and its recent application to parasitic flatworms [67–69] and roundworms [70, 71], it is now feasible to consider hookworms as a molecular foundry by which to constitutively deliver foreign molecules such as antibodies, small molecules, or even extracellular vesicles containing defined payloads), to the gut. Before the notion of transgenic hookworms can be considered in practical terms, wild-type hookworm culture methods, protocols, and regulatory challenges need to be addressed.

### Safety of experimental hookworm infection

Experimental human hookworm infection has been performed on over 200 participants in the modern era, mostly using the Kar Kar Island isolate; in many cases, repeat infections have been undertaken (Table 1). No deaths have been reported from experimental hookworm infection, and no serious adverse events have been reported in contemporary studies. A wide range of doses have been administered, ranging from 3 to 1,000 L3 per dose. It is intuitive that higher doses (>50 larvae per dose) may result in an increased frequency of adverse events, including more severe dermal reactions and gastrointestinal symptoms. While the safety of lower doses is established, data on the adverse event profile of higher doses are sparse. Only 8 studies have reported the use of more than 100 larvae per dose [20,26–28,40,72–74], and since 1978, only 3 individuals have been received doses of 100 L3 [32,47,57], with all other participants receiving a maximum of 50 L3 per dose.

### Secondary infective complications

As production of hookworm larvae relies on coproculture, it is intuitive that hookworm larvae may be contaminated with human pathogens and may cause infection when penetrating the skin (see below for a discussion on methods to reduce this bioburden).

In 1918, Malvoz and Lambient provided experimental proof that hookworm larvae penetrating skin may carry pathogens. In their experiments, sputum heavily contaminated with *Mycobacterium tuberculosis* was smeared onto the shaved belly skin of 2 guinea pigs. To one animal, a drop of liquid containing numerous hookworm larvae was added, the other animal serving as a control. The animal inoculated with both larvae and *M*. *tuberculosis* died within a month of disseminated tuberculosis, while the control animal remained healthy. The experiment was then repeated using *Bacillus anthracis* as the contaminant, and similar results were observed [75].

There has only been a single report of secondary bacterial infection complications secondary to experimental hookworm infection. Kawanishi made this report in 1932, reporting a case of suppurative axillary lymphadenitis requiring surgical drainage following dermal inoculation of 700 *N*. *americanus* larvae to the forearm. *Escherichia coli* was cultured from the wound material and the volunteer eventually made a full recovery [76].

Cline and colleagues were first to document donor screening for infectious pathogens in 1984 [62]. Screening of donors now includes exclusion of blood-borne viruses (HIV, hepatitis B virus (HBV), and hepatitis C virus (HCV)), faecal bacterial and viral pathogens, and exclusion of colonisation with organisms with selected antibiotic resistance phenotypes, such as extended spectrum beta-lactamase (ESBL) gram-negative organisms and methicillin-resistant *Staphylococcus aureus* (MRSA) [61]. Detailed screening procedures for donors of faecal material have been developed to facilitate faecal microbiota transplantation. These standardised criteria would also be relevant for hookworm faeces donation [77].

### Dermal symptoms

The development of a pruritic erythematous rash at the site of larval application is universal, with the development of a blistering eruption described in more severe cases. Cline and colleagues described skin eruptions occurring in 29 of 30 participants, with the individual in whom no reaction occurred later admitting to manipulating the dressing [62]. As few as 3 larvae reliably produced the dermal reaction [33]. Dermal symptoms persist for 1 to 4 weeks depending on severity.

Sensitisation, with resulting augmentation of the dermal reaction, has been described after repeated exposure. Brumpt describes this phenomenon in a case series of 51 patients who received doses of approximately 400 *A*. *duodenale* larvae per inoculation for treatment of polycythaemia. The first inoculation resulted in an erythematous, pruritic rash developing after 24 hours and persisted for 10 days. In 9 cases, repeat inoculation was performed, which resulted in the immediate development of an urticarial rash at the site of inoculation, lasting for a few weeks. A total of 4 patients received an additional dose of larvae resulting in an intense urticarial reaction with localised oedema and induration [40]. Similarly, following the primary inoculation, creeping eruption was observed in repeat exposures [73]. In this respect, it is curious that such dermal reactions are uncommonly reported in hookworm endemic settings where exposure would continue lifelong.

### Respiratory symptoms

Although hookworm infection is commonly cited as a cause of Loeffler syndrome (eosinophilic pneumonitis), there is little experimental evidence to support this. Brumpt provides a detailed account of respiratory symptoms following dermal inoculation with large doses (400 larvae) of *A*. *duodenale*. The onset of retrosternal chest pain, dry hacking, nonproductive cough and dysphonia, with a nocturnal predominance commences from the fourth day after inoculation, persisting for up to 3 weeks. On physical examination, there is erythema of the pharynx, but signs of pulmonary consolidation are absent. Chest X-rays performed daily from day 3 were unremarkable [40].

Lee and colleagues confirmed these findings when using mixed inoculations of 150 to 800 *A*. *duodenale* and *A*. *ceylanicum* larvae. Symptoms occurred from day 7 and lasted 2 weeks with unremarkable chest X-ray findings [27]. Unlike Loeffler syndrome, eosinophilia is not present, crepitation is not heard on chest auscultation, and chest X-ray does reveal abnormality [40]. Symptoms correlate with the ascent of larvae into the trachea, at which point larvae may be isolated from sputa [26].

Severe pharyngitis, bronchitis, and haemoptysis have been reported after dermal inoculation of 700 *N*. *americanus* larvae [72], and similarly, a hacking cough with nocturnal predominance, red bleeding pharynx and aphonia, were reported after inoculation with 400 larvae [40]. Diemert and colleagues observed mild respiratory symptoms in 2 of 10 participants who received 25 larvae and 7 of 10 who received 50 larvae; however, there was no placebo-controlled comparator in this study [52]. Significantly, in a pair of randomised, placebo-controlled trials specifically assessing respiratory symptoms following inoculation with 10 *N*. *americanus* L3, respiratory symptoms related to inoculation were not observed [45,46].

### Gastrointestinal symptoms

Gastrointestinal symptoms including abdominal pain, diarrhoea, flatulence, nausea, and vomiting have been commonly reported, with some individuals reporting nocturnal predominance or periprandial associations. The typical onset of symptoms is in the fourth week (range 3 to 9 weeks), with symptoms persisting for 2 to 4 weeks [40,74].

In contrast to the sensitisation observed in dermal reactions, habituation to intestinal colonisation by adult hookworms with repeated inoculation is well described [32,40,41,74]. Brumpt described gastrointestinal symptoms after doses of 400 *A*. *duodenale* in 53 participants as “acute duodenitis lasting 15 days with diarrhoea lasting 2 to 4 weeks.” Amelioration of symptoms with repeated doses of *A*. *duodenale* larvae was observed [40]. In a study exploring the humoral responses to experimental *N*. *americanus* infection, Ogilvie and colleagues performed repeated infections in a volunteer with 250 *N*. *americanus* L3. Each infection was treated at approximately day 90, 28 days prior to repeat infection. The first experiment resulted in “severe gastrointestinal disturbance” characterised by nausea, pain, and diarrhoea commencing at day 25 and continuing to day 70. A similar but milder syndrome occurred after the second inoculation, while gastrointestinal symptoms were absent in the third and fourth inoculations. Faecal egg counts at day 90 remained comparable between infections [74].

### Iron deficiency anaemia

IDA is well described in naturally acquired hookworm infections. Clinically significant anaemia is limited to individuals with high parasite burdens, low iron reserves, and insufficient dietary iron intake. Additionally, the species of hookworm is important, with *A*. *duodenale* associated with greater blood loss and increased likelihood of IDA than infection with *N*. *americanus* because of the wasteful feeding habits of the ancylostomatids [2].

As noted above, experimentally induced *A*. *duodenale* infection has been used to manage polycythaemia [39], with a summary reporting clinical experience in over 50 subjects in 1952 [40].

Secondary IDA has also been described in 10 participants inoculated via gelatine capsule with 150 *A*. *duodenale* L3. Mean haemoglobin concentrations reduced from 130 (range 120 to 150)g/L to 104 (98 to 110) g/L over 3 months [27]. There has been one case of experimentally induced IDA after infection with 1000 *N*. *americanus* larvae, reported in 1932 by Kawanishi [72].

Importantly, IDA has not been described as a consequence of experimental human infection in any contemporary clinical trials reported since 1969 [32,38,41,42,45–54,56–58,62,63,73,74,78,79].

### Hypereosinophilia

A case report describes the development of eosinophilic myocarditis in an individual who infected themself with larvae (believed to be *N*. *americanus)* purchased online for treatment of their asthma and allergic rhinitis. Dermal application of larvae was performed on 3 occasions with 35, 50, and 50 larvae, respectively, first inoculation 9 months prior and the third inoculation 7 weeks prior to presentation. The subject was found to have a peripheral eosinophil count of 10 × 10^9^/L, which resolved after treatment with albendazole [80]. No other reports of symptomatic hypereosinophilia could be identified with either natural or experimental hookworm infection.

### Rescue medication

Although dermal symptoms from hookworm inoculation are typically benign, pruritus at the penetration site may be distressing and may be more severe in the setting of repeated inoculation. Topical corticosteroids may be administered to reduce local pruritus and inflammation. Although albendazole is effective in treating intestinal infection, its larvicidal activity against *N*. *americanus* is limited [62]. While ivermectin is effective in treating cutaneous larvae migrans from zoonotic hookworm infection [81], its effectiveness in mitigation of dermal symptoms from human larvae has not been studied.

Gastrointestinal symptoms following experimental human infection are unpredictable and may be severe. Of 244 participants experimentally administered *N*. *americanus* larvae in contemporary trials (Table 1), 9 individuals have been reported to require early termination of infection due to acute hookworm-related gastrointestinal symptoms. In a dose ranging study, severe symptoms were noted only in the 2 subjects who received higher doses of *N*. *americanus* (100 and 50 larvae, respectively), while only mild symptoms were experienced at doses of fewer than 50 L3 [47]. While this suggests that the gastrointestinal symptoms may be dose related, the occurrence of severe symptoms is not strictly dose related, with 2 participants reported to require termination of infection after application of just 10 larvae [45,46]. In a phase 1b randomised controlled trial investigating therapeutic hookworm infection for coeliac disease, 2 participants experienced severe gastrointestinal symptoms requiring termination of infection after inoculation with 20 larvae, while those receiving 40 larvae reported only mild and tolerable symptoms [50]. Albendazole is effective at eliminating the intestinal stages of infection and gastrointestinal symptoms resolve within 24 to 48 hours of treatment.

### Preparation of inocula

#### Isolation of Hookworm ova

Historically, hookworm ova were obtained by dissection of female worms (presumably recovered from faeces after anthelmintic drug treatment) [26,35], from the faeces of infected patients [27,41,42,82], from laboratory hamsters [45–47] and, most commonly, from infected volunteer donors [29,32,48,50–54,57,58,62,63,72,79].

The use of experimentally infected volunteers as hookworm egg donors was first reported by Payne in 1923 [29]. As noted above, the majority of contemporary trials have been facilitated by the use of *N*. *americanus* larvae that were originally sourced from Papua New Guinea and maintained in human donors [43].

### Larval culture

Culture methods, postculture larval processing and storage, and estimation of viability have been sparsely described in detail. Larvae are produced by mixing hookworm eggs with charcoal or vermiculite, using various modifications of the Harada–Mori method [51–53,83]. Although some authors have reported pretreatment of faeces with antifungal and antibacterial agents prior to culture [50–52], the role of antimicrobials at this stage has not been established. The addition of charcoal would be expected to inactivate antibiotic present in the preparation [84], and the development of hookworm larvae has been shown to be dependent on the presence of intestinal bacterial flora [3]. Larval harvest is performed after 7 to 10 days incubation at 25 to 28°C, after which larvae are suspended in an aqueous solution.

The Immune Modulation Research Group (IMRG) (Nottingham University, UK) provided the first description of current Good Manufacturing Practice (cGMP)-compliant production of hookworm larvae [52].

Methods to produce larvae that do not rely on coproculture have been reported [3], although never used for larval preparation for clinical trials. Culture methods free of faeces in which eggs are separated from the faeces and disinfected prior to culture would represent an attractive option for clinical trials in reducing the bioburden associated with larval preparations. However, the methods to achieve this are more time consuming and labour intensive and may inhibit scale up for use in clinical trials.

### Larval bioburden

As larvae are produced by coproculture, and the presence of feacal microflora has been shown to be essential for larval development [3], it is intuitive that infective larvae be both colonised and contaminated with elements of the human microbiome. In 1957, Harada described a decontamination method for producing larvae that entailed washing larvae in antiseptic solution. Significant overgrowth of bacteria occurred by 14 days in the absence of antibiotic (streptomycin) added to the storage solution. Furthermore, it was observed that the addition of antibiotic resulted in a toxic effect on the larvae [85].

In accordance with regulations, recent trials performed in the USA and EU have had assessment of microbial bioburden performed on the supernatant of harvested hookworm solutions [48,52]. In both cases modified, Harada–Mori culture was performed, and the harvested larvae were then washed a number of times in sterile water [52] or incubated in antiseptic solution and then washed [48]. Both trials report that the larval solution met regulatory requirements for bioburden at the time of preparation. Although it would be expected that bacterial regrowth would occur over time, it is unclear if bioburden was analysed at the time of use.

### Larval characterisation

The use of living organisms as an investigational product presents complexities in terms of reproducibility of production of the challenge agent. Unlike sterile investigational products, the attributes of living organisms may vary over time, from batch to batch and be affected by changes in procedure, handling, and environment. These attributes require characterisation and controlling for use clinical experimental models.

### Larval identification and speciation

The identity of larvae produced from a human donor pool was confirmed by PCR or microscopic evaluation [48,50–52] to be *N*. *americanus* prior to release for use in the recent clinical trials. This step maybe particularly important if donors who may have been exposed to other helminths by travel to or residence in areas where intestinal nematode infections are prevalent.

## Larval infectivity and viability

Of central importance to the use of hookworm larvae as an investigational product is their infectivity and ability to survive somatic migration and mature to adults in the gut.

Although the age of larvae at the time of inoculation has been shown to influence infectivity [29,30], this variable has been infrequently reported. Variability in the age of hookworm larvae used in recent human studies may account for the discordant results demonstrated in recent controlled trials in hookworm-naive participants. Hoogerwerf and colleagues demonstrated 100% patency and robust faecal egg counts (>500 epg) after inoculation with 50 L3 used within 10 days of production [48,51], whereas Diemert and colleagues reported patent infection in only 40% and 90% of participants who received 25 and 50 larvae, respectively, and with modest faecal egg output at 16 to 166 epg. These larvae had been imported to Washington, DC from the UK, and, although the authors stated that larvae batches were 80% motile at the time of use, the age of the larvae was not stated.

Similarly, 9 of 40 participants failed to develop patent infection in a recent multicentre clinical trial [50]. Significantly, 6 of the participants who failed to develop patent infection were at an international trial site where larvae were imported for the trial. Age of larvae at time of use was not detailed in this trial.

Estimates of viability have rarely been reported, with observed larval motility the only reported methodology [48,50–52]. As larval motility varies throughout the day and according to environmental conditions, a thermally induced motility assay is required for reliable results [86]. However, motility may be a poor surrogate estimate of infectivity, and the quality of movement and larval condition is probably also important. The larvae used by Croese and colleagues had been transported from the UK to Australia and were approximately 6 weeks old when used. Of 2,000 larvae dispatched, only 306 (15.3%) were motile on arrival in Australia [32].

### Clinical end points for human trials

#### Adverse events

Adverse event monitoring, grading, assessment of causality, and expectedness is mandated in interventional clinical trials. In order to compare adverse event profiles across trials, a standardised approach is required. The FDA supplies guidance on toxicity grading for volunteers enrolled in vaccine clinical trials [87]. This includes a standardised grading scale for assessment of dermal symptoms associated with the injection site and has been adapted for use in 2 clinical trials [49,52].

#### Parasitological

Confirmation and quantification of successful establishment of intestinal infection after inoculation are vital for interpretation of experimental results. Estimation of successful infection following dermal application of hookworm larvae can be achieved by a range of means, including assessing dermal reactions and penetration efficiency, the emergence of peripheral blood eosinophilia, faecal examination for helminth eggs and Charcot–Leyden crystals (CLC), and the expulsion of adult worms after anthelmintic treatment, coproculture, qPCR for parasite DNA, or capsule endoscopy.

#### Dermal assessment

As previously described, dermal itch is a reliable sign that the larvae have penetrated the skin. An estimation of the penetration efficiency by a dermally applied inoculum of larvae may be performed by examining the inoculation site using a magnification glass. Discrete punctate haemorrhages are visible at the site of penetration between 48 and 72 hours postadministration. Although this method has been used in several experimental studies, it remains semiquantitative as general erythema at the site of dermal penetration may obscure the individual penetration sites [23,26,28–30,33,37,62,63,76].

#### Peripheral blood eosinophilia

Peripheral blood eosinophilia is universally reported, with detectable rises in eosinophil counts from as early as day 7 (mean day 21), with peak values measured between the fifth and sixth weeks [24,27,29,31,32,34,37–39,41,42,45–47,53,54,57,62,63,72–74,79].

#### Faecal microscopy

Microscopic examination of faeces for hookworm eggs is a convenient and semiquantitative method for confirming intestinal infection [88] and includes well established and validated methodologies, including formol-ether concentration (FEC), McMaster technique, FLOTAC and mini-FLOTAC, and the Kato–Katz direct smear [89]. Faecal egg counts have been used to assess efficacy of anthelmintic chemotherapy, although the utility of so-called faecal egg count reduction test (FECRT) is modest at best [90], and a gold standard for diagnosis and quantification of hookworm burden is lacking [91]. Considerable day-to-day variation in faecal egg count is observed, particularly in controlled human hookworm infection where parasite burden is low [51]. Furthermore, without prompt preservation or refrigeration of faecal samples, the sensitivity of microscopy for detection of *N*. *americanus* eggs diminishes significantly within hours of defecation [92,93].

Of the 35 published trials, 23 reported using faecal microscopy to confirm patent infection [23,24,26–33,38,41,42,45–47,62–64,72–74,82]. A total of 16 participants who failed to develop patent infection (as assessed by microscopy) were from 5 studies, 3 of which used relatively low numbers of larvae (10 to 15 larvae total) [45,46,54] and in the remaining 2 studies larvae were shipped internationally, which may have resulted in suboptimal viability [50,52].

CLC are formed following eosinophil degranulation and are composed of eosinophil lysophospholipase. The crystals can be detected in the faeces of individuals with intestinal helminth infection [94,95]. In 2 studies where examination was performed for CLC, they were present in all patent infections [32,57].

#### Coproculture

Coproculture has been demonstrated to be more sensitive than microscopy for diagnosis of *Strongyloides stercoralis* and hookworm infection [96] and has been used to confirm patency of infection with *N*. *americanus* in microscopy-negative participants in clinical trials [45,46]. Furthermore, interventions that result in either reduced fertility of adult worms or fitness in successive generations would be highly relevant in community eradication programmes but may not be reflected in established diagnostic modalities.

#### Detection of hookworm nucleic acid

Molecular estimation of faecal hookworm DNA content by quantitative polymerase chain reaction (qPCR) assays has greater sensitivity than microscopy. Faecal hookworm DNA concentration has been correlated with faecal eggs per gram and interpolation of faecal epg from faecal DNA concentration has been validated at ranges of 250 to 6,000 epg [97]. However, at low faecal egg density, estimation of parasite burden may be confounded by other issues such as density-dependent worm fecundity and the maturation of embryonated eggs with variable numbers of nuclei.

Recent clinical studies have employed qPCR to confirm the establishment of intestinal colonisation and to infer intensity of infection [50,52,53,83]. Hoogerwerf and colleagues performed paired Kato–Katz and quantitative PCR assays on samples collected from 23 participants who were inoculated with 50 to 150 larvae. The qPCR cycle threshold was found to correlate with egg counts by Kato–Katz. Diemert and colleagues also used paired samples to compare McMaster floatation egg counts to qPCR with faecal hookworm DNA concentration interpolated to epg. qPCR estimation of epg was higher than that determined using McMaster [52].

#### Endoscopy

Estimation of intestinal worm count in dogs by capsule endoscopy after experimental infection has been demonstrated to closely correlate with the number of worms recovered at necroscopy [98]. In humans, endoscopy has been employed in 3 clinical trials. This allows direct observation of the intestinal worms and can confirm successful infection in participants who fail to develop patent infections (as assessed by microscopy) [54]. Additionally, bleeding spots may be visible from hookworm feeding. Capsule endoscopy is a convenient and user-friendly method that has been validated in hookworm infection, although there is some interobserver variation [32]. Conventional endoscopy has the advantage of providing opportunities for biopsy of intestinal mucosa [53,54].

## Conclusions

Experimental human hookworm infection is not novel and has been demonstrated to be safe in clinical trials across the 20th century. Controlled human hookworm infection and challenge studies provide an opportunity to better understand the host–parasite relationship, the underlying pathobiology, and presents the opportunity to test interventions both directed at reducing hookworm disease and harnessing the immunomodulatory effects of hookworm infection.

However, to improve the fidelity of experimental models and results, better characterisation of hookworm larvae as an investigational product must be pursued. Descriptions of culture methodologies, postharvest processing of larvae, estimation of viability, and age at time of use should be reported.

The challenges in experimental hookworm research in the 21st century can only be met with improvements in the basic science. In 1955, Harada and Mori published “A new method for culturing hookworm,” which remains the methodology in use today [99]. A cGMP method for developing hookworm without the requirement to culture faeces from human donors and a methodology to store the larvae while maintaining viability, such as cryopreservation is required.

**Fig 2:**
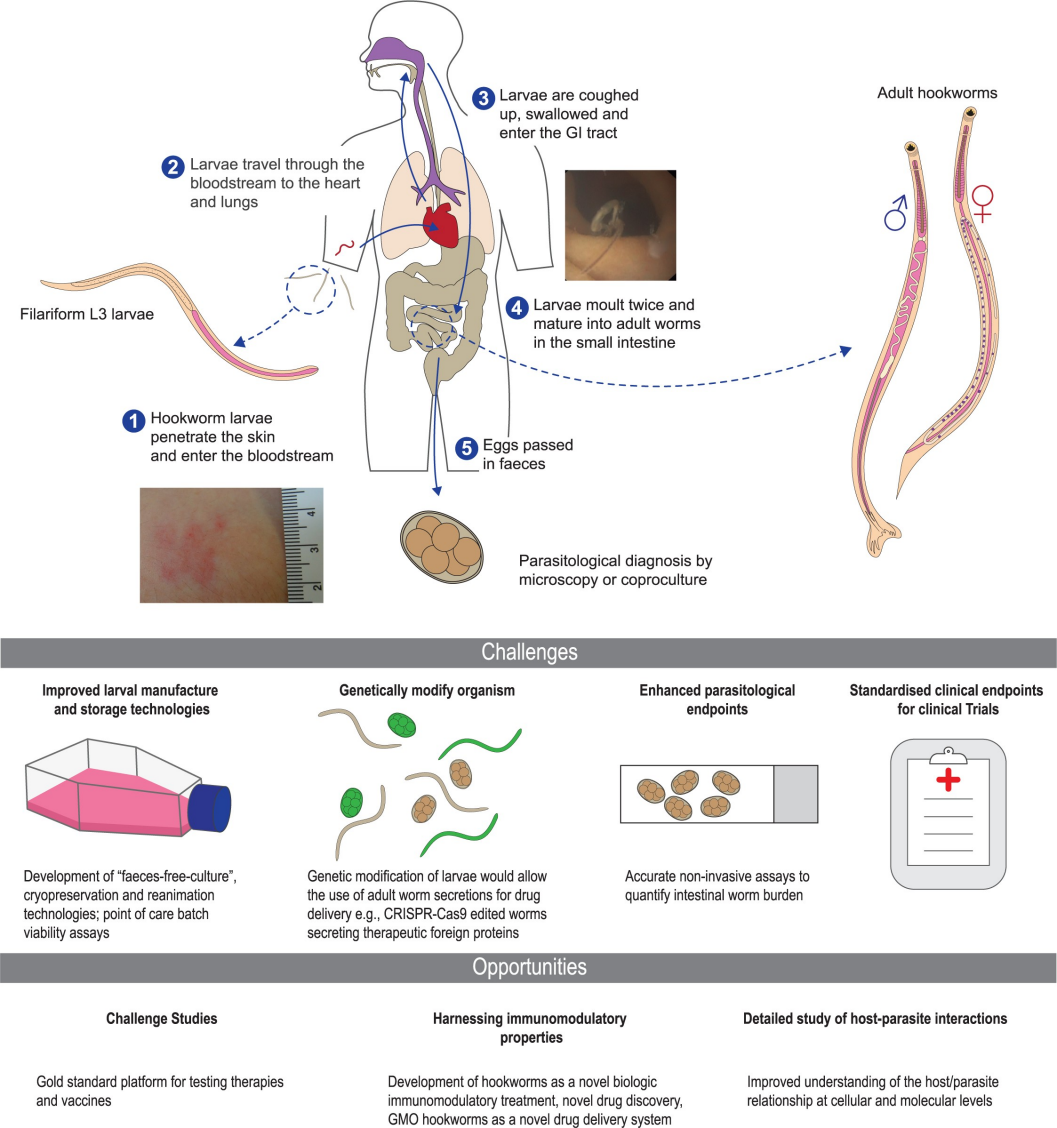
Challenges and opportunities in experimental human hookworm infection. GI, gastrointestinal.

Likewise, our estimation of the intensity of intestinal infection is based on surrogate markers such as faecal egg counts and faecal hookworm DNA concentration and are imprecise. To facilitate assessment of the therapeutic potential of hookworms as immunomodulators or as novel vectors of drug delivery, or the effectiveness of vaccines and other interventions, an accurate assessment of the number of hookworms present in the intestine is required (Fig 2).

**Key Learning Points**

- Dermal reaction is predictable and occurs with as few as 3 larvae, and gastrointestinal symptoms are unpredictable and may occur with as few as 10 larvae.
- There have been no deaths or serious adverse events from contemporary experimental hookworm infection and the pathology associated with natural hookworm infection is not demonstrated in experimental infection. Symptoms of hookworm infection are reliably relieved by anthelmintic medication.
- Characterisation of culture methods, postharvesting processing and of hookworm larvae, including age at time of use, are essential for producing reproducible results.
- Development of noncoproculture, cGMP larval production, and storage methodologies are required for continued human experimental infection.
- Improvements to parasitological end points are needed. While microscopy is well established, it is less sensitive than molecular methods, although no gold standard exists.

**Top Five Papers**

1. Loukas A, Hotez PJ, Diemert D, Yazdanbakhsh M, McCarthy JS, Correa-Oliveira R, et al. Hookworm infection. Nat Rev Dis Primers. 2016;2:16088.
2. Diemert D, Campbell D, Brelsford J, Leasure C, Li G, Peng J, et al. Controlled Human Hookworm Infection: Accelerating Human Hookworm Vaccine Development. Open Forum Infect Dis. 2018;5(5):ofy083-ofy. doi: 10.1093/ofid/ofy083
3. Hoogerwerf MA, Koopman JPR, Janse JJ, Langenberg MCC, van Schuijlenburg R, Kruize YCM, et al. A randomized controlled trial to investigate safety and variability of egg excretion after repeated controlled human hookworm infection. J Infect Dis. 2020. Epub 2020 Jul 10. doi: 10.1093/infdis/jiaa414. PubMed PMID: 32645714.
4. Mortimer K, Brown A, Feary J, Jagger C, Lewis S, Antoniak M, et al. Dose-ranging study for trials of therapeutic infection with Necator americanus in humans. Am J Trop Med Hyg. 2006;75(5):914–20.
5. Chapman PR, Webster R, Giacomin P, Llewellyn S, Becker L, Pearson MS et al. Vaccination of human participants with attenuated Necator americanus hookworm larvae and human challenge in Australia: a dose-finding study and randomised, placebo-controlled, phase 1 trial. Lancet Infect Dis. 2021. doi: 10.1016/S1473-3099(21)00153-5

## Supporting information

S1 Table Studies included in this narrative review. (DOCX)
