# Supplementary material for: Experimental human hookworm infection: a narrative historical review
Source: PLoS Negl Trop Dis. 2021 Dec 9;15(12):e0009908. doi: 10.1371/journal.pntd.0009908 (PMC8659326; doi:10.1371/journal.pntd.0009908)
Supplement: S1 Table — (DOCX) [file pntd.0009908.s001.docx]

**S1 Table. Summary of the included experimental hookworm infection trials.**

| **1^st^ Author (year)** | **Study design** | **Participants (n)** | **Inoculum number (cumulative dose)** | **Notable outcomes** |
| --- | --- | --- | --- | --- |
| Looss.  (1898) [1] | Natural experiment - laboratory accident. Dermal application followed by faecal examination for eggs | 1 | Unknown | Theory that hookworm infection develops via dermal penetration by larvae |
| Looss.  (1901) [2] | Experimental infection Dermal application of larvae prior to limb amputation, microscopic and histopathologic examination | 1 | Unknown | Confirmation of skin penetration theory |
| Bentley.  (1902) [3] | Experimental infection, dermal application of contaminated soil vs sterilised soil, natural history | 4 | Unknown | Confirmation that larvae present in contaminated soil are the cause of “Ground Itch” and confirmation of infection via dermal penetration |
| Payne.  (1923) [4] | Experimental infection, larvae of various ages | 3 | 12-81 Na | Failure of infection with old larvae; probable immune response to repeated infection |
| Svensson. (1927) [5] | Experimental infection, comparison of infectivity of *Ancylostoma* vs *Necator* larvae | 2 | 25 Ad / Na | Superior efficiency of Necator infection via dermal route |
| Shelmire.  (1928) [6] | Experimental infection, dermal inoculation, description of AE | 18 | 100 Ab | CLM in 16 of 18 participants |
| Kawanashi. (1932) [7] | Experimental infection, natural history | 3 | 150-700 (450-1100) Na | Anaemia and suppurative lymphadenitis requiring hospitalisation in 1 participant (1100 larvae) |
| Maplestone.  (1933) [8] | Experimental infection  Dermal inoculations of *A. duodenale, A. caninum, A. brazilience* (India) and *N. americanus.* Description of AE | 51 | 10-30 | Minimal dermal reaction *A. braziliense* (India), prompting reclassification of A. brazilience found in the New World (now *A. ceylanicum*) |
| Kendrick.  (1934) [9] | Experimental infection, dermal and oral inoculations, natural history | 25 | 200-300 (200-700) Na / Ad | Description of egg outputs and duration of infection. Confirmation of inability of *N. americanus* to infect by oral route |
| Palmer.  (1941) [10] | Experimental infection, natural history | 1 | 50 Na | Longitudinal analysis of faecal egg content |
| Brumpt.  (1952) [11] | Case series. Therapeutic use, for treatment of polycythaemia and hypertension | 53 | 300-700 Ad | The first report of therapeutic infection. Demonstration of hookworm related anaemia. Description of sensitisation to dermal and habituation to gastrointestinal symptoms |
| Beaver.  (1955) [12] | Experimental infection, inoculation with 3 larvae to estimate individual worm output | 9 | 3 Na | 5/9 participants developed patent infection and all experienced dermal reaction following only 3 larvae |
| Mizuno.  (1963) [13] | Experimental infection, inoculation by dermal and oral infections | 129 | 8-1258 Na / Ad | Superior infectivity of *N. americanus* through dermal infection with no infection via oral infection |
| Yasudo.  (1964) [14] | Experimental infection, oral inoculation | 6 | "Numerous" Na / Ad | *N. americanus* unable to produce patent infection through oral inoculation |
| Wijers.  (1965) [15] | Dermal infection with *A. braziliense* var *ceylanicum* | 8 | 50-150 Ac | Confirmation of patent infection, dermal inoculation of *A. braziliense* var *ceylanicum.* CLM absent |
| Lee.  (1967) [16] | Experimental infection, oral inoculation, investigation of AE | 10 | 150 Ad | Wakana syndrome described and reduction in haemoglobin of 20g/L by end of study |
| Ball.  (1969) [17] | Experimental infection Repeated inoculation of author, humoral immune response | 1 | 300, 100, 100, 25, 25, 25, 25, 25, 25 Na | Description of humoral immune responses to experimental infection |
| Areekaul.  (1970) [18] | Dermal inoculation *with A. ceylanicum* and *A. caninum* | 2 | 35 Ac, 800 Ac / *A. caninum* | Confirmed infection by dermal inoculation with *A. ceylanicum* |
| Areekaul.  (1973) [19] | Experimental infection via oral inoculation | 3 | 2000 Ac/*A. caninum*, 130 Ac | Patent infection was not produced after oral inoculation |
| Ogilvie.  (1978) [20] | Experimental infection Repeated inoculation of author, humoral immune response | 1 | 250, 250, 250, 250 Na | Description of habituation to GI AE with repeated exposure |
| Cline.  (1984) [21] | Placebo controlled RCT, efficacy, albendazole | 29 | 45 Na | The first RCT. Limited larvicidal effect of albendazole, even when taken with a fatty meal |
| Carrol.  (1986) [22] | Experimental infection, investigation of natural history | 2 | 1200 Ac | No dermal reaction noted despite the development of gastroenterological symptoms and patent infection |
| White.  (1986) [23] | Experimental infection, investigation of CMI | 3 | 50 Na | Description of eosinophilic response to *N americanus* infection |
| Maxwell.  (1987) [24] | Experimental infection Description of humoral response | 5 | 50 Na | Detailed description of adverse events, 1 of 5 required rescue medication for GI AE |
| Landmann.  (2003) [25] | Experimental infection, comparison or oral and dermal inoculation | 1 | 100-200 *A. caninum* | Demonstration of eosinophilic enteritis, patent infection was not observed |
| Wright.  (2005) [26] | Experimental infection, HNV. Observation of immune responses | 1 | 50 + 50 Na | GI AE noted following the primary infection were not observed with repeat infection |
| Croese.  (2006) [27] | Experimental infection, as therapy for Crohn's disease and HNV to become HW egg donors. | 12 | 50-100 Na | Landmark study investigating therapeutic infection for immuno-modulation. First reported use of larvae originating from Kar Kar island maintained in human donors at Nottingham University |
| Croese.  (2006) [28] | Experimental infection, as therapy for Crohn's disease | 5 | 25-150 Na | Characterisation of mucosal response to hookworm feeding. First study to use endoscopy to confirm infection |
| Mortimer.  (2006) [29] | Experimental infection of HNV - dose ranging study | 10 | 10-100 Na | Detailed descriptions of adverse effects. 2 participants required rescue medication for GI symptoms (100 and 50 L3) |
| Geiger.  (2008) [30] | Experimental infection of HNV | 2 | 50 Na | Detailed immunological investigations following experimental infection |
| Feary.  (2009). [31] | Placebo controlled RCT, as therapy for allergic rhinitis | 15 | 10 Na | Rescue medication required in 1 subject for GI AE.  Respiratory AE not observed |
| Feary.  (2010) [32] | Placebo controlled RCT, as therapy for asthma | 17 | 10 Na | Rescue medication required in 1 subject for GI symptoms.  Respiratory AE not observed |
| Daveson.  (2011) [33] | Placebo controlled RCT, as therapy for coeliac disease | 10 | 10, 5 (15) Na | No terminations of infection, patent infection confirmed with endoscopy |
| Croese.  (2015) [34] | Open label trial, as therapy for coeliac disease | 12 | 10, 10 (20) Na | Well tolerated, no termination of infection required  Demonstration of therapeutic effect |
| Diemert.  (2018) [35] | Experimental infection HNV. Observation of AE and egg outputs | 20 | 25-50 (25 / 50) Na | Lower than expected patency after inoculation  cGMP manufactured larvae imported from UK |
| Hoogerwerf.  (2019) [36] | Experimental infection of HNV. Description of AE and egg outputs, natural history | 4 | 50 Na | Analysis of egg output over time.  Description of AE. |
| Croese. (2020) [37] | Placebo control phase 1b RCT, as therapy for coeliac disease | 47 | 10,10 (20) or 20,20 (40) Na | Unexpectedly high failure of infection after shipping of larvae, 2 participants required rescue medication for GI AE |
| Tanasecu. (2020) [38] | Placebo controlled, phase 2 RCT, as therapy for relapsing multiple sclerosis. | 35 | 25 | cGMP larvae, only 65.7% patency |
| Hoogerwerf. (2021) [39] | Placebo controlled, phase 1 RCT. Description of AE and egg outputs, natural history | 23 | 50 / 50,50 / 50,50,50 | Rescue medication required in 3 participants with GI AE. Definitive description of faecal egg output |
| Chapman. (2021) [40] | Placebo controlled phase 1b RCT. Vaccination with live-attenuated larvae. | 15 | 30 | Detailed description of adverse events. 100% patency but discordant hatchability of eggs.  No rescue medication required. |
|  | **Total participants** | **599** |  |  |

Abbreviations: Ac Ancylostoma ceylanicum, Ad *A. duodenale*, AE adverse event, cGMP current good manufacturing practice, CMI cell mediated immunity, CLM cutaneous larva migrans, GI gastrointestinal, HNV hookworm naïve volunteer, HW hookworm, Na Necator americanus, RCT randomised controlled trial.

Inoculum dose – dose range indicated by values separated by hyphen, repeated doses separated by comma, cumulative dose in brackets.

1. Looss A. Zur Lebensgeschichte des Ankylostoma duodenale. Centralblatt Bakteriol Parasitol Infekt. 1898;24:441–9, 83–8.

2. Looss A. On the penetration of Ancylostoma larvae into the human skin. Centralblatt Backteriol Parasitenkunde. 1901;29:733-9.

3. Bentley CA. On the causal relationship between "ground itch," or "pani-ghao," and the presence of the alrvae of the *Ankylostoma duodenale* in the soil. British medical journal. 1902;1(2143):190-3. Epub 1902/01/25. PubMed PMID: 20760011; PubMed Central PMCID: PMCPMC2511788.

4. Payne FK. Investigations on the Control of Hookworm Disease. XXXI. The Relation of the Physiological Age of Hookworm Larvae to their Ability to Infect the Human host. American Journal of Hygiene. 1923;3(5):584.

5. Svensson RM. Notes on Diferences in Activity and Resistance between the Larvae of Ancylostoma duodenale and Necator americanus. The Journal of parasitology. 1927;13(3):203-5.

6. Shelmire B. Experimental creeping eruption from a cat and dog hookworm (A.Braziliense). Jama. 1928;91(13):938-44. doi: doi:10.1001/jama.1928.02700130016005.

7. Kawanishi K. Experimental studies of the morphological changes of the blood in percutaneous infections with Necator americanus of man. Jour Med Assn Formosa. 1932;31(19 (English summary)).

8. Maplestone PA. Creeping Eruption Produced by Hookworm Larvae. Indian Medical Gazette. 1933;68(5):251-7.

9. Kendrick JF. The Length of Life and the Rate of Loss of the Hookworms, Ancylostoma Duodenale and Necator Americanus. American Journal of Tropical Medicine and Hygiene. 1934;14(s1):363-79.

10. Palmer ED. The Course of The Daily Egg Output During An Early INfection With The Hookworm Necator Americanus. Am J Epidemiol. 1941;34(1):1-12.

11. Brumpt LC. [Clinical deductions based on fifty cases of induced ancylostomiasis]. Ann Parasitol Hum Comp. 1952;27(1-3):237-49. Epub 1952/01/01. PubMed PMID: 14944070.

12. Beaver PC. Observations on Necator infections resulting from exposure to three larvae. Revista Iberica de Parasitologia. 1955:713-21.

13. Mizuno T, R. Y. Studies on the infection route of hookworms with reference to experimental infection in human hosts with larvae of Ancylostoma duodenale and Necator americanus. Jap JHyg). 1963;18(4):311+35.

14. Yasudo K. Studies on the infection mode of hookworms. Experimental infection in human hosts with the infective larvae of Necator americanus or the larvae of Ancylostoma duodenale soaked in human defibrinated blood. Nihon eiseigaku zasshi Japanese journal of hygiene. 1964;19:285-309. Epub 1964/12/01. PubMed PMID: 14247848.

15. Wijers DJ, Smit AM. Early symptoms after experimental infection of man with Ancylostoma braziliense var. ceylanicum. Tropical and geographical medicine. 1966;18(1):48-52. Epub 1966/03/01. PubMed PMID: 6006057.

16. Lee M, Kim J, D., Lee J, K. Studies on Ancylostomiasis. Korean J Nucl Med. 1967;1(1):55-66.

17. Ball PA, Bartlett A. Serological reactions to infection with Necator americanus. Transactions of the Royal Society of Tropical Medicine and Hygiene. 1969;63(3):362-9. PubMed PMID: 4893921.

18. Areekul S, Radomyos P, Viravan C. Experimental infection of Ancylostoma ceylanicum in man. Journal of the Medical Association of Thailand = Chotmaihet thangphaet. 1970;53(3):190-4. Epub 1970/03/01. PubMed PMID: 5423051.

19. Areekul S, Viravan C, Radomyos P. Experimental infection of A. ceylanicum by oral route in man. The Southeast Asian journal of tropical medicine and public health. 1973;4(1):138. Epub 1973/03/01. PubMed PMID: 4736947.

20. Ogilvie BM, Bartlett A, Godfrey RC, Turton JA, Worms MJ, Yeates RA. Antibody responses in self-infections with Necator americanus. Transactions of the Royal Society of Tropical Medicine and Hygiene. 1978;72(1):66-71. PubMed PMID: 635980.

21. Cline BL, Little MD, Bartholomew RK, Halsey NA. Larvicidal activity of albendazole against Necator americanus in human volunteers. The American journal of tropical medicine and hygiene. 1984;33(3):387-94. Epub 1984/05/01. PubMed PMID: 6731670.

22. Carroll SM, Grove DI. Experimental infection of humans with Ancylostoma ceylanicum: clinical, parasitological, haematological and immunological findings. Tropical and geographical medicine. 1986;38(1):38-45. Epub 1986/03/01. PubMed PMID: 3961908.

23. White CJ, Maxwell CJ, Gallin JI. Changes in the structural and functional properties of human eosinophils during experimental hookworm infection. The Journal of infectious diseases. 1986;154(5):778-83. Epub 1986/11/01. PubMed PMID: 3021866.

24. Maxwell C, Hussain R, Nutman TB, Poindexter RW, Little MD, Schad GA, et al. The clinical and immunologic responses of normal human volunteers to low dose hookworm (Necator americanus) infection. The American journal of tropical medicine and hygiene. 1987;37(1):126-34. Epub 1987/07/01. PubMed PMID: 3605493.

25. Landmann JK, Prociv P. Experimental human infection with the dog hookworm, Ancylostoma caninum. The Medical journal of Australia. 2003;178(2):69-71. Epub 2003/01/16. PubMed PMID: 12526725.

26. Wright V, Bickle Q. Immune responses following experimental human hookworm infection. Clinical and experimental immunology. 2005;142(2):398-403. Epub 2005/10/20. doi: 10.1111/j.1365-2249.2005.02945.x. PubMed PMID: 16232230; PubMed Central PMCID: PMCPMC1809522.

27. Croese J, O'Neil J, Masson J, Cooke S, Melrose W, Pritchard D, et al. A proof of concept study establishing Necator americanus in Crohn's patients and reservoir donors. Gut. 2006;55(1):136-7. Epub 2005/12/14. doi: 10.1136/gut.2005.079129. PubMed PMID: 16344586; PubMed Central PMCID: PMC1856386.

28. Croese J, Wood MJ, Melrose W, Speare R. Allergy controls the population density of Necator americanus in the small intestine. Gastroenterology. 2006;131(2):402-9. Epub 2006/08/08. doi: 10.1053/j.gastro.2006.05.019. PubMed PMID: 16890593.

29. Mortimer K, Brown A, Feary J, Jagger C, Lewis S, Antoniak M, et al. Dose-ranging study for trials of therapeutic infection with Necator americanus in humans. The American journal of tropical medicine and hygiene. 2006;75(5):914-20. Epub 2006/11/25. PubMed PMID: 17123987.

30. Geiger SM, Fujiwara RT, Santiago H, Correa-Oliveira R, Bethony JM. Early stage-specific immune responses in primary experimental human hookworm infection. Microbes and infection / Institut Pasteur. 2008;10(14-15):1524-35. Epub 2008/10/14. doi: 10.1016/j.micinf.2008.09.003. PubMed PMID: 18848637.

31. Feary J, Venn A, Brown A, Hooi D, Falcone FH, Mortimer K, et al. Safety of hookworm infection in individuals with measurable airway responsiveness: a randomized placebo-controlled feasibility study. Clinical and experimental allergy : journal of the British Society for Allergy and Clinical Immunology. 2009;39(7):1060-8. Epub 2009/04/30. doi: 10.1111/j.1365-2222.2009.03187.x. PubMed PMID: 19400893; PubMed Central PMCID: PMCPMC2728895.

32. Feary JR, Venn AJ, Mortimer K, Brown AP, Hooi D, Falcone FH, et al. Experimental hookworm infection: a randomized placebo-controlled trial in asthma. Clinical and experimental allergy : journal of the British Society for Allergy and Clinical Immunology. 2010;40(2):299-306. Epub 2009/12/25. doi: 10.1111/j.1365-2222.2009.03433.x. PubMed PMID: 20030661; PubMed Central PMCID: PMCPMC2814083.

33. Daveson AJ, Jones DM, Gaze S, McSorley H, Clouston A, Pascoe A, et al. Effect of hookworm infection on wheat challenge in celiac disease--a randomised double-blinded placebo controlled trial. PloS one. 2011;6(3):e17366-e. doi: 10.1371/journal.pone.0017366. PubMed PMID: 21408161.

34. Croese J, Giacomin P, Navarro S, Clouston A, McCann L, Dougall A, et al. Experimental hookworm infection and gluten microchallenge promote tolerance in celiac disease. The Journal of allergy and clinical immunology. 2015;135(2):508-16 e5. doi: 10.1016/j.jaci.2014.07.022. PubMed PMID: 25248819.

35. Diemert D, Campbell D, Brelsford J, Leasure C, Li G, Peng J, et al. Controlled Human Hookworm Infection: Accelerating Human Hookworm Vaccine Development. Open forum infectious diseases. 2018;5(5):ofy083-ofy. doi: 10.1093/ofid/ofy083.

36. Hoogerwerf MA, Coffeng LE, Brienen EAT, Janse JJ, Langenberg MCC, Kruize YCM, et al. New Insights Into the Kinetics and Variability of Egg Excretion in Controlled Human Hookworm Infections. The Journal of infectious diseases. 2019;220(6):1044-8. Epub 2019/05/12. doi: 10.1093/infdis/jiz218. PubMed PMID: 31077279.

37. Croese J, Miller GC, Marquart L, Llewellyn S, Gupta R, Becker L, et al. Randomized, Placebo Controlled Trial of Experimental Hookworm Infection for Improving Gluten Tolerance in Celiac Disease. Clin Transl Gastroenterol. 2020;11(12):e00274. Epub 2021/01/30. doi: 10.14309/ctg.0000000000000274. PubMed PMID: 33512796; PubMed Central PMCID: PMCPMC7678792.

38. Tanasescu R, Tench CR, Constantinescu CS, Telford G, Singh S, Frakich N, et al. Hookworm Treatment for Relapsing Multiple Sclerosis: A Randomized Double-Blinded Placebo-Controlled Trial. JAMA Neurology. 2020;77(9):1089-98. doi: 10.1001/jamaneurol.2020.1118.

39. Hoogerwerf MA, Koopman JPR, Janse JJ, Langenberg MCC, van Schuijlenburg R, Kruize YCM, et al. A Randomized Controlled Trial to Investigate Safety and Variability of Egg Excretion After Repeated Controlled Human Hookworm Infection. The Journal of infectious diseases. 2021;223(5):905-13. Epub 2020/07/10. doi: 10.1093/infdis/jiaa414. PubMed PMID: 32645714.

40. Chapman PR, Webster R, Giacomin P, Llewellyn S, Becker L, Pearson MS, et al. Vaccination of human participants with attenuated Necator americanus hookworm larvae and human challenge in Australia: a dose-finding study and randomised, placebo-controlled, phase 1 trial. The Lancet infectious diseases. 2021. Epub 2021/08/23. doi: 10.1016/S1473-3099(21)00153-5. PubMed PMID: 34419209.
